# Niche-Partitioning of Edaphic Microbial Communities in the Namib Desert Gravel Plain Fairy Circles

**DOI:** 10.1371/journal.pone.0109539

**Published:** 2014-10-03

**Authors:** Jean-Baptiste Ramond, Annelize Pienaar, Alacia Armstrong, Mary Seely, Don A. Cowan

**Affiliations:** 1 Center for Microbial Ecology and Genomics (CMEG), Genomic Research Institute, University of Pretoria, Pretoria, South Africa; 2 Gobabeb Research and Training Center (GRTC), Walvis Bay, Namibia; Missouri University of Science and Technology, United States of America

## Abstract

Endemic to the Namib Desert, Fairy Circles (FCs) are vegetation-free circular patterns surrounded and delineated by grass species. Since first reported the 1970's, many theories have been proposed to explain their appearance, but none provide a fully satisfactory explanation of their origin(s) and/or causative agent(s). In this study, we have evaluated an early hypothesis stating that edaphic microorganisms could be involved in their formation and/or maintenance. Surface soils (0–5cm) from three different zones (FC center, FC margin and external, grass-covered soils) of five independent FCs were collected in April 2013 in the Namib Desert gravel plains. T-RFLP fingerprinting of the bacterial (16S rRNA gene) and fungal (ITS region) communities, in parallel with two-way crossed ANOSIM, showed that FC communities were significantly different to those of external control vegetated soil and that each FC was also characterized by significantly different communities. Intra-FC communities (margin and centre) presented higher variability than the controls. Together, these results provide clear evidence that edaphic microorganisms are involved in the Namib Desert FC phenomenon. However, we are, as yet, unable to confirm whether bacteria and/or fungi communities are responsible for the appearance and development of FCs or are a general consequence of the presence of the grass-free circles.

## Introduction

**Figure 1:**
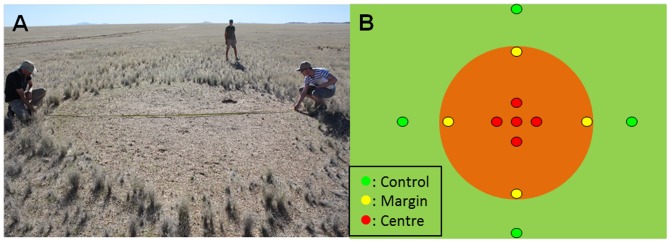
Fairy Circle sampling. Photograph of a Fairy Circle in the gravel plains of the Namib Desert (A) with the schematic of the sampling strategy employed (B).

The Namib Desert is unique in harboring the enigmatic Fairy Circles (FC) (or Fairy Rings). They occur and have been studied predominantly in the sand dune environment of the eastern Namib but also occur on the gravel plains [1], [2]. FCs are circular vegetation-free patterns (generally between 2 to 12 m in diameter), surrounded by grass species of the genus *Stipagrotis* (Figure 1A). Their distribution is restricted to a region in the Pro-Namib zone (60 to 120 km inland), from southern Angola to northern South Africa [2]. The Namib Desert FCs have been described as ‘living organisms’, as they appear (birth), enlarge (growth) and ultimately disappear (death), with an estimated life span of around 60 years [3].

Since first being reported in the 1970's, many studies and hypotheses have attempted to explain the origins and lifestyles of FCs (most reviewed in [2]): these hypotheses include local radioactivity [4], insect (termite/ant) activities [5]–[11], the release of volatile chemicals (e.g. allelopathic compounds by dead *Euphorbia damarana* plants [12], semi-volatile products from termite-related activities [8], [13] or hydrocarbon-liked compounds of geochemical origins [14]), or plant spatial self-organization [15], [16]. The allelopathic compound and radioactivity hypotheses have already been refuted in field studies [2]. The ‘abiotic gas leakage’ and ‘grass harvesting social insect (ant/termite)’ hypotheses were contested, based on mathematical modelling (e.g. remote sensing, spatial pattern analysis and vegetation modelling) [16]. The results obtained by Getzin and colleagues [16] suggested that FC distribution was indicative of self-organized models, i.e. in agreement with the ‘spatial self-organization’ hypothesis [15]. The authors nevertheless noted that more factors should be implemented (e.g. plant root system and soil moisture data) to fully validate the hypothesis. The current trend to include microbiological data when modeling edaphic ecosystems [17], [18] may be even more critical in arid edaphic environments where most ecosystem processes are microbially-mediated [19]. It must also be noted that gravel plain FCs are largely unstudied ([1] and [3] only, to our knowledge) compared to the eastern Namib FCs. The patchiness of gravel plain FCs (as shown in Figure 1A) is not consistent with the self-organization pattern hypothesis [15], [16].

Despite the fact that the potential role of microorganisms as contributing agents of FC development has not yet been tested experimentally, it has been previously discussed because of circumstantial support for the involvement of microbial processes. Significantly higher alkene emissions have been observed from FC soils than from the vegetated surroundings soils [14]; and alkene evolution has been shown to be indicative of anaerobic microbially-mediated alkane reduction [20]. Using Namib FC soils, bioassay experiments suggested phytotoxicity [2] and germination studies suggested the involvement of a biologically active abiosis factor leading to plant decay [8]. An ‘*in situ*’ pot trial also suggested the possible involvement of either a semi-volatile compound or a (microbial?) toxin in plant-growth inhibition [13].

The current study was initiated with the working hypothesis that edaphic microorganisms could be significant agents in the formation and/or maintenance of Namib Desert FCs. The experimental basis of this study was a comprehensive sampling of surface soils from within FCs (centre and margin) and from external vegetated (controls) sites. Unlike most previous FC studies, which have focused in FC sites situated in the dune sands to the east of the Namib Sand Sea, we selected FCs in the Namib Desert gravel plains. Our analytical approach included T-RFLP fingerprinting of bacterial (16S rRNA gene) and fungal (ITS region) phylogenetic markers and soil chemical analyses (10 different variables), using multivariate statistical analyses to cross-correlate the experimental data.

## Materials and Methods

### Fairy Circle soil sampling and storage

Namib Desert Fairy Circle soils were sampled under the permits N° P0063476, permit for the importation of controlled goods from the Department of Agriculture, Forestry and Fisheries of the Republic of South Africa, and N° ES 29529, permit for single consignment export of minerals from the Ministry of Mines and Energy of the Republic of Namibia.

Surface soils (0–5 cm; approx. 200g per sample) from five independent Fairy Circles were collected in the gravel plains of Namibia (−23°31′37.09”S/15°11.15”E) near the Gobabeb Research and Training Center (http://www.gobabebtrc.org/) in April 2013, following a strategy shown schematically in Figure 1. A total of thirteen samples per FC were collected: four control soils from outside the FCs and nine within each FCs; 4 at the circle margin and 5 in the center. The samples were stored at 4°C during transport to the CMEG Laboratory (University of Pretoria, South Africa). One gram subsamples were stored at −80°C for subsequent molecular analyses and the residual soil at 4°C for chemical analyses.

### Soil Chemistry analyses

Soil chemistry analyses were conducted at the Soil Science Laboratory of the University of Pretoria (South Africa) according to standard quality control procedures [21]. All solutions and reagents used were supplied by Merck Chemicals (South Africa) and, apart for pH measurements, soil samples were sieved (2 mm) prior to analysis.

The slurry technique was used to measure pH recorded with a pH meter (Crison basic 20, Barcelona, Spain) by mixing 4 g of soil with 10 ml of deionized water and allowing it to settle for 1h. The Walkey-Black method [22] was used to determine soil total organic carbon (C) content with minor modifications. 10 mL of 1M potassium dichromate solution was added to 2 g of soil. 10 mL of sulfuric acid (96%) was then added, and the mixture was left to cool at room temperature for 30 min. 150 mL deionized water and 10 mL concentrated (96%) orthophosphoric acid was then added to the mixture which was left to cool at room temperature for 30 min. 1 mL phenylalanine was added and titration was performed by adding iron(II) ammonium sulphate solution until the reaction endpoint was reached; i.e. when the solution changed from purple to green.

Exchangeable ammonium (NH_4_^+^) and nitrate (NO_3_^−^) soil content was determined by steam distillation as described [23] with minor modifications. 5 g of soil was mixed with 50 mL of a 2M KCl solution and shaken for 30 min at 220 rpm. Samples were then left to settle for 1 min and the supernatant was filtered through a 110 mm diameter Whatman n°2V filter paper and stored overnight at 4°C. The addition of 0.2 g MgO to the filtrate liberated ammonium, and the residual nitrate was determined by the reduction to nitrite (NO_2_) *via* the addition of ∼ 2g Devarda alloy powder [24].

Total organic P was determined by the P Bray method described by [25] with minor modifications. 50 mL of P Bray-1 solution was added to 4 g of soil. The mixture was hand-shaken for exactly 1 min prior to filtration using 110 mm diameter Whatman n°2V filter paper. The phosphorous concentration of the filtrate was then determined by Inductively Coupled Plasma-Optical Emission Spectroscopy (ICP-OES) (Spectro Genesis, Germany).

Total ion concentrations were determined by adding 40 mL of a 0.2M ammonium acetate solution to 4 g of soil. The mixture was shaken for 1 hour and the supernatant filtered through 110 mm diameter Whatman n°2V filter paper. 15 mL of the filtrated were used to determine the concentrations of iron (Fe), calcium (Ca), potassium (K), magnesium (Mg) and sodium (Na) by ICP-OES.

### Metagenomic DNA extraction

Total soil DNA was extracted from 0.3 g samples using the Powersoil DNA isolation kit according to the manufacturer's instructions (MOBIO laboratories, San Diego, USA). DNA concentrations were estimated with a NanoDrop spectrophotometer (NanoDrop Technologies, Montchanin, DE, USA).

### PCR amplification, purification and restriction digestion

All polymerase chain reactions (PCRs) were carried out in a Bio-Rad Thermocycler (T100 TM Thermal Cycler). Bacterial 16S rRNA encoding genes were amplified using the universal bacterial primers 341F (5′-CCTACGGGAGGCAGCAG-3′)/908R (5′- CCGTCAATTCMTTTGAGTTT -3′) [26] and the Fungal ITS regions amplified using the universal primer set ITS1 (5′-CTTGGTCATTTAGAGGAAGTAA-3′)/ITS4 (5′-TCCTCCGCTTATTGATATGC-3′) [27]. PCR was carried out in 50 µl reaction volumes, where each reaction contained 1X PCR buffer, 0.2 U DreamTaq polymerase (Fermentas, USA), 200 µM of each dNTP, 0.5 µM of each primer, 4% bovine serum albumin (BSA), 2% to 6% DMSO to increase PCR specificity (the concentration used was sample-dependent) and between 5 and 20 ng of metagenomic DNA.

16S rRNA gene PCR amplifications were carried out as follows: 5 min at 95°C for denaturation; 25 cycles of 30 s at 94°C, 30 s annealing at 54°C and 105 s at 72°C; and a final elongation step of 7 min at 72°C. Fungal ITS region PCR amplifications were performed as follows: 5 min at 95°C for denaturation; 25 cycles of 1 min at 94°C, 50 s annealing at 55°C and 105 s at 72°C; and a final elongation step of 7 min at 72°C.

To perform T-RFLP analyses, the 341F and ITS1 primers were 5′-end FAM-labelled and the PCR products were purified using the GFX TM PCR DNA and gel band purification kit as directed by the supplier (GE Healthcare, UK). 200 ng of purified PCR products for 16S rRNA gene T-RFLP were digested with the restriction enzymes *Msp*I at 37°C overnight, while 400 ng of purified ITS amplicons was digested overnight with *Hae*III.

### Terminal-Restriction Fragment Length Polymorphism (T-RFLP) analyses

T-RF size was determined by capillary electrophoresis using a Applied Biosystems DNA Sequencer 3130 (Applied Biosystems, Foster City, California, USA) and according to the molecular weight standard GeneScan-600LIZ V2 (Applied Biosystem), with an error of ±1 bp. Individual T-RFs were considered as Operational Taxonomic Units (OTUs), with recognition that each OTU may comprise more than one distinct bacterial ribotype. Peak height was used to identify each T-RF and characterize their relative abundance in the total T-RFLP profiles, which was used as a proxy for OTU abundance in the microbial populations.

### Statistical analyses

Multivariate analyses of T-RFLP and environmental data were performed using the software Primer 6 (Primer-E Ltd, UK). Valid T-RF peaks (between 30 and 567 bp for 16S rRNA gene T-RFLP or 30 and 800 bp for ITS region T-RFLP) from triplicate T-RFLP profiles were identified, compiled and aligned to produce large data matrices using the online software T-REX (http://trex.biohpc.org/) [28]. The community structures obtained were analyzed by ordination using non-metric multidimensional scaling (nMDS) of Bray-Curtis similarity matrices of square-root transformed data. Two-way crossed analysis of similarity (ANOSIM) tests were used to assess significant differences in the structure of assemblages among the Fairy Circles sampled and between the different sampling ‘zones’ (centre/margin/control vegetated soils) [29]. Multivariate dispersion (MVDISP) was used to measure ‘within-zone’ assemblage dispersion [30]. For each FC, one-way ANOSIM was used to test for differences in microbial community assemblages in the three FC-zones.

Prior to principal component analysis (PCA), the environmental variables were analyzed using a Draftsman plot [31] to evaluate the need for transformations, i.e. any skewness in the dataset. Total Carbon (% C) and ammonium (NH_4_^+^) were initially log(x +1) transformed, and the complete environmental data set was normalized to perform PCA. A resemblance matrix based on Euclidean distances was created using the normalized set prior to two-way crossed ANOSIM.

## Results and Discussion

Plant-microbe interactions actively shape plant diversity by various processes (reviewed in [32]), and soil pathogens have been described as active drivers of vegetation-succession [32]. As Namib Desert FCs are circular vegetation-free patterns surrounded by a healthy vegetation-covered matrix, we have hypothesized that a soil-born plant pathogen could be a causative agent of the phenomenon. This would not be unique: a fungus (*Sclerotinia homoeocarpa*) has been shown to be responsible for a plant disease, known as the Dollar Spot, which is morphologically similar to the FC phenomenon. *S. homoeocarpa* infects turfgrass species and, at a much smaller scale (i.e., the size of a dollar coin), creates FC-like circular necrotic patches that grow with time [33]. We therefore investigated the potential involvement of both edaphic bacterial and fungal communities in the origin and/or maintenance of the Namib Desert gravel plain FCs, as members from both phylogenetic groups are known to exhibit phyto-toxicity (e.g., [34]-[37]).

The measurement of ten edaphic chemical variables (Table 1), analyzed by PCA, provided no clear evidence of an “FC zonation effect” (Figure S1); i.e., the composite soil chemistries of the samples from the three zones tested (FC center/FC margin/external vegetated control) were randomly scattered on the PCA plot. Two-way crossed ANOSIM confirmed this observation with (marginally significant) global R values <0.2, indicating that the different sampling zones and each FC could not clearly be separated [38]. Van Rooyen and colleagues [2] observed a similar trend with no differences between FC microhabitat soil chemistries in each of the sandy FC sites studied. Contrastingly, clear FC-specific patterns of hydrocarbon-like gas emissions were observed in similar FCs [14] which led to the geochemical origin hypothesis of Namib Desert FCs. Taken together, these results could suggest that gas emission rather than different soil chemistries could play a direct, or indirect, role in FC formation or maintenance.

**Table 1:** Characterizations of the Fairy Circles sampled and of their edaphic chemical properties.

Fairy Circle	Dimensions (mxm)	Area (m^2^)	Zone	% Carbon	NH_4_^+^ (mg N/kg)	NO_3_^−^ (mg N/kg)	P (mg/kg)	Fe (mg/kg)	Ca (mg/kg)	K (mg/kg)	Mg (mg/kg)	Na (mg/kg)	pH
FC1	4.6×3.7	13.75	Control	0.52(±0.91)	9.19(±2.16)	8.47(±2.54)	13.24(±1.75)	17.52(±4.84)	162.95(±50.98)	17.11(±0.62)	5.81(±0.49)	0.95(±0.39)	8.16 (±0.13)
			Margin	0.88(±1.54)	13.66(±4.82)	9.88(±3.73)	16.79(±1.26)	14.91(±2.47)	177.54(±50.2)	18.42(±2.53)	6.08(±0.31)	1.39(±0.46)	8.07 (±0.08)
			Centre	1.02 (±2.21)	10.3(±1.71)	10.54(±4.24)	16.01(±3.1)	15.72(±1.32)	138.63(±11.83)	14.24(±3.71)	4.85(±0.46)	2.34(±1.34)	8.03 (±0.18)
FC2	3.6×3.2	9.05	Control	ND	33.51(±44.33)	12.86(±1.38)	13.00(±1.80)	16.89(±0.68)	132.54(±18.56)	14.21(±2.86)	5.17(±0.5)	0.83(±0.57)	8.13 (±0.1)
			Margin	0.64 (±0.79)	18.04(±10.19)	12.04(±2.45)	11.90(±2.97)	13.11 (±6.76)	263.06 (±118.21)	16.72 (±1.78)	6.04 (±0.61)	2.24 (±1.65)	8.15 (±0.09)
			Centre	0.11(±0.17)	12.29(±2.19)	12.06(±2.10)	12.72(±0.66)	11.35(±3.97)	193.39(±42.68)	14.23(±1.16)	5.68(±0.27)	1.13(±0.68)	8.09 (±0.13)
FC3	5×3.7	14.53	Control	0.01(±0.03)	12.91(±0.6)	13.76(±1.80)	13.46(±1.34)	18.12(±3.49)	211.71(±115.24)	15.75(±1.95)	5.89(±1.04)	1.43(±1.70)	8.05 (±0.13)
			Margin	1.84(±3.67)	9.8(±2.85)	12.23(±3.00)	14.62 (±1.56)	17.76 (±3.89)	161.14 (±39.01)	14.13(±2.26)	5.32 (±0.38)	0.91 (±0.37)	8.19 (±0.05)
			Centre	0.18(±0.38)	8.91(±3.04)	9.00(±1.97)	14.04(±1.44)	17.53(±2.05)	158.67(±20.20)	14.04(±1.52)	5.38(±0.14)	0.74(±0.07)	8.05 (±0.09)
FC4	3.6×4.2	14.7	Control	0.10(±0.20)	8.50(±1.30)	6.50(±1.00)	13.91 (±1.87)	19.59(±4.38)	131.86(±29.77)	14.26(±1.34)	5.69(±0.25)	0.53(±0.20)	8.22 (±0.07)
			Margin	0.03(±0.06)	7.06(±0.76)	6.63(±1.22)	14.61(±1.62)	17.85(±2.25)	165.94(±41.3)	13.49(±1.49)	5.06(±0.23)	0.63(±0.18)	8.06 (0.07)
			Centre	ND	8.63(±2.34)	8.91(±3.30)	13.01(±1.1)	15.12(±3.72)	202.83(±102.74)	14.02(±1.46)	5.70(±0.33)	0.76(±0.25)	8.09 (±0.09)
FC5	3.8×3.1	9.25	Control	ND	6.16(±0.87)	6.79(±0.23)	14.79(±1.87)	14.2(±4.62)	191.01(±80.12)	18.43(±3.40)	5.43(±0.17)	1.37(±0.33)	8.11 (±0.09)
			Margin	ND	11.6(±6.54)	9.08(±0.66)	13.65(±1.33)	17.01(±6.26)	179.58(±54.02)	14.34(±0.70)	5.95(±0.25)	0.72(±0.09)	8.13 (±0.14)
			Centre	ND	7.03(±0.88)	8.58(±2.30)	12.26(±2.18)	15.34(±5.73)	243.54(±91.70)	14.04(±0.84)	6.26(±0.38)	0.88(±0.15)	8.11 (±0.15)

Values are given as mean ± SD. ND: Not determined as below detection limit.

**Figure 2:**
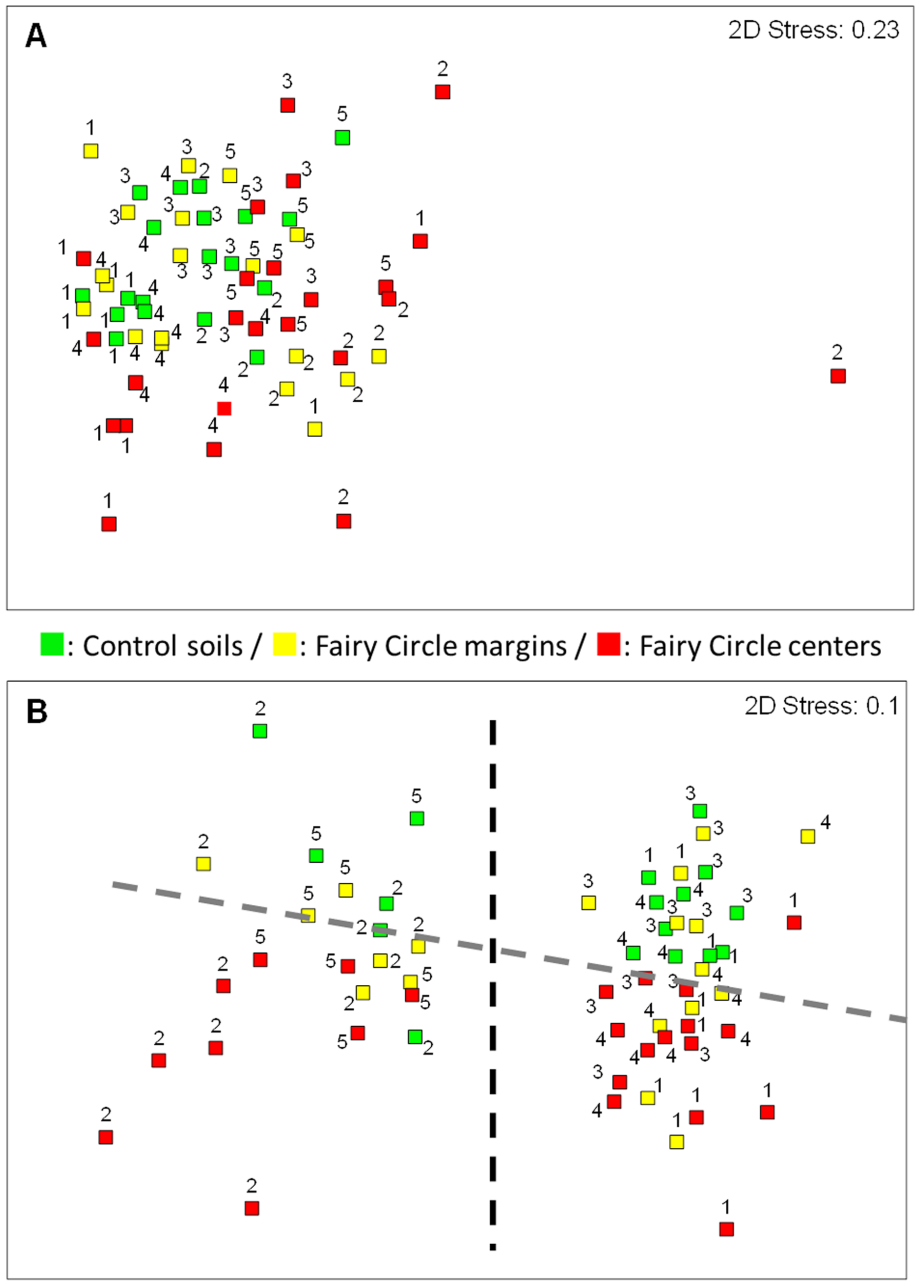
Two dimension nonmetric multidimensiobal scaling (2D-nMDS) plot of Bray–Curtis similarity of fungal (A) and bacterial (B) community structures based on ITS region and 16S rRNA gene square-root transformed T-RFLP profiles respectively. Numbers refer to the respective Fairy Circles. The dashed lines indicate virtual separations between group of samples (Grey: control vs FC centre communities/Black: FC2 and FC5 vs FC1, FC3 and FC4).

The nMDS plots showing the ordination of the fungal community structures revealed by T-RFLP suggested a similar trend (Figure 2A); i.e., samples from each FC zones were randomly positioned (2D-stress> 0.2). Contrastingly, the nMDS ordination of the bacterial community T-RFLP fingerprints indicated some discrimination between communities from the FC centre and the control soils (virtually separated by the dashed grey line in Figure 2B), and between communities from different FCs (i.e., FC2 and FC5 communities appeared distinct from those of FC3, FC4 and FC5: virtually separated by the dashed black line in Figure 2B). Two-way crossed ANOSIM (Table 2) showed strongly significant differences in the structure of the bacterial and fungal assemblages between the different FC zones (particularly the control and FC-center bacterial communities: R = 0.702; p = 0.01). The simplest explanation for these results would be habitat filtration due to the absence of rhizosphere and their associated microbial assemblages within the non-vegetated FC soils (e.g., [39], [40]). However, highly significant differences between the microbial assemblages of each individual FC were also detected (ANOSIM R>0.9 in 6 of the 10 FC pairwise comparisons, and p consistently <0.05; Table 2). Following this observation, the bacterial and fungal community fingerprint of each FC was analyzed separately by ANOSIM, independently testing for differences in their respective FC zones (Table 3). Excluding the fungal community from FC 2, these analyses showed that both the control and FC-centre communities (pairwise comparisons) and/or the communities from the three zones (global test) were significantly different (p <0.05). These results suggest that each independent FC houses a significantly distinct surface edaphic microbial community which is significantly different from that of the vegetation-covered control soils, and strongly suggest that the absence of rhizosphere/rhizospheric communities cannot satisfactorily explain the structure differences in edaphic microbial communities observed between FCs and external soils.

**Table 2:** Results of two-way crossed ANOSIM tests based on Bray-Curtis similarity matrices from square-root transformed bacterial and fungal T-RFLP profiles.

		Differences among Fairy Circle zones			
		Bacterial communities		Fungal communities	
		R	p	R	p
Global Test		0.418	0.001 *	0.26	0.001 *
Control vs Margin		0.103	0.1	0.26	0.001 *
Control vs Centre		0.702	0.001 *	0.329	0.001 *
Margin vs Centre		0.401	0.001 *	0.234	0.007 *
		**Differences among Fairy Circles**			
		**Bacterial communities**		**Fungal communities**	
		R	p	R	p
Global Test		0.688	0.001 *	0.477	0.001 *
FC 1 vs FC 2		0.994	0.001 *	0.461	0.002 *
FC 1 vs FC 3		0.466	0.001 *	0.599	0.001 *
FC 1 vs FC 4		0.237	0.004 *	0.234	0.023 *
FC 1 vs FC 5		0.993	0.001 *	0.581	0.001 *
FC 2 vs FC 3		0.969	0.001 *	0.429	0.002 *
FC 2 vs FC 4		0.958	0.001 *	0.522	0.001 *
FC 2 vs FC 5		0.214	0.054	0.274	0.004 *
FC 3 vs FC 4		0.454	0.001 *	0.593	0.001 *
FC 3 vs FC 5		1	0.001 *	0.686	0.001 *
FC 4 vs FC 5		1	0.001 *	0.736	0.001 *

R: ANOSIM statistic; p: probability level. *: Significantly different (p <0.05).

**Table 3:** One-way ANOSIM statistics comparing the bacterial and fungal community structures the predefined zones of each FC studied.

		Bacterial communities		Fungal communities	
Fairy Circle	ANOSIM	R	p	R	p
FC1	Global test	0.245	0.057	0.247	0.014 *
	Control vs Centre	0.446	0.036 *	0.281	0.048 *
	Margin vs Centre	0.15	0.198	0.244	0.095
	Control vs Margin	0.185	0.229	0.365	0.029 *
FC2	Global test	0.463	0.001 *	0.115	0.104
	Control vs Centre	0.763	0.008 *	0.163	0.103
	Margin vs Centre	0.663	0.008 *	(-)0.094	0.881
	Control vs Margin	(-)0.083	0.771	0.375	0.086
FC3	Global test	0.511	0.002 *	0.364	0.007 *
	Control vs Centre	0.763	0.008 *	0.400	0.048 *
	Margin vs Centre	0.675	0.008 *	0.488	0.024 *
	Control vs Margin	(-)0.104	0.771	0.188	0.143
FC4	Global test	0.463	0.001 *	0.245	0.04 *
	Control vs Centre	0.788	0.008 *	0.313	0.071
	Margin vs Centre	0.244	0.063	0.206	0.103
	Control vs Margin	0.365	0.029 *	0.25	0.2
FC5	Global test	0.408	0.025 *	0.423	0.015 *
	Control vs Centre	0.714	0.067	0.679	0.133
	Margin vs Centre	0.167	0.2	0.407	0.086
	Control vs Margin	0.333	0.2	0	0.6

R: ANOSIM statistic; p: probability level. *: Significantly different (p <0.05).

Recent satellite data have demonstrated that the Namib Desert Fairy Circles could be considered as ‘living organisms’, as they appeared (birth), grew and disappeared (death) [3]. It was concluded that any study attempting to unravel the origins of FCs should take into account the fact that they are not static environments but vary over time [3], [16]. The underlying corollary from this observation is that individual FCs within a site in the Namib Desert (e.g. such as those in our study which were all sampled within one hectare area) might be at different stages in their ‘lifespan’ and potentially constitute different biotopes (e.g., re-vegetated ‘ghost’, newly forming or matured/ing FCs; [3]). If we assume that the surface soil microbial communities are not static throughout the lifespan of a FC (i.e., change in parallel with the evolution of the FC biotope; [39], [41]), we are provided with a possible explanation for the differences observed between individual FC microbial communities (Table 2).

Distinct ‘drivers’ or environmental disturbances could also lead to alternative FC edaphic communities. For example, fluctuating hydrocarbon-like gas seepages [14], the action of different phytopathogenic microorganisms/pathovars [34] or dissimilar toxin(s) infection stages [37] could all lead to different FC biotopes within a single site. The microbial-mediated hypotheses are supported by the fact that two fungal T-RFs (OTUs 246 and 683; data not shown) were consistently observed within the margin and/or center of all five FCs studied, but never in the external control samples and could therefore constitute molecular signatures of “FC-related” fungi [33].

To test whether FC microbial communities were more heterogeneous than those of the vegetated control soils, multivariate dispersion indices were calculated (MVDISP routine in Primer6; [30]). The MVDISP index was lowest for the control communities and highest for the FC-centre communities, for both the bacterial (MVDISP Control 0.854 <MVDISP FC Margin 0.987 <MVDISP FC Centre 1.08) and fungal (MVDISP Control 0.659 <MVDISP FC Margin 0.92 <MVDISP FC Centre 1.261) communities. As a higher MVDISP index is indicative of higher multivariate dispersal, this clearly demonstrates that the vegetated (control) soil microbial communities exhibited lower intra-variability (or higher homogeneity) than the FC communities; the FC centre communities being the more variable, i.e. the more heterogeneous. These intra-variability differences could be explained by the adaptation of the ‘intra-FC’ (margin and centre) microbial communities to environmental disturbance leading to their stochastic assembly [41].

**Figure 3:**
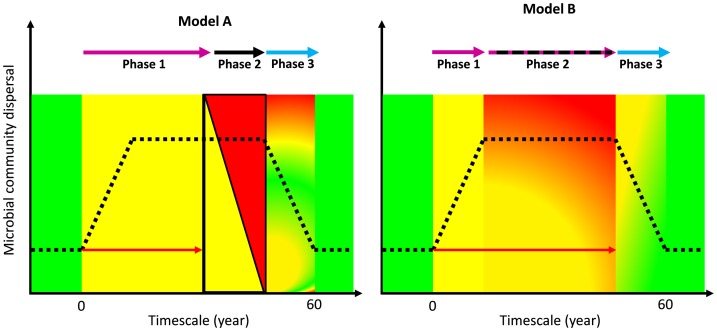
Models hypothesizing microbial community assembly in Namib Desert Fairy Circles. Arrow indicate the assembly processes (purple: stochastic/black: niche-partitioning/blue: neutral). Colors are represent virtually the FC zones (Green: vegetated covered control soils/Yellow: FC margins/Red: FC Centers) where these processes occur. Red arrows indicate the origin in time and length of the environmental disturbance responsible for FC appearance. The x-axis does not reflect proportionally the time scale.

Based on the observation that FCs appear to evolve over time [3], we suggest that the control vegetation-covered soils constitute a primary environmental state (or precursor), the FC margins represent a transitional phase, and the centre non-vegetated soils an alternative environment. By applying the three phase community assembly model after disturbance defined by Ferrenberg and colleagues [41] and based on the principles of microbial community dispersal, we propose two models to potentially explain the assembly of Namib Desert gravel plain Fairy Circle microbial communities (Figure 3).

We suggest that an environmental disturbances (e.g., the effect of active microbial phyto-pathogens, (dis)continuous gas leakage or plant spatial self-organization as a response to resource scarcity; [14], [33], [42]) could lead to plant necrosis which would disrupt the homogenous precursor (control) edaphic communities. Subsequently, FC-margin communities would further develop by stochastic assembly processes (phase 1; Models A and B; Figure 3). Where the disturbance is not continuous in time, the microbial communities of the three FC zones would develop based on niche-partitioning; i.e., vegetation-covered *versus* vegetation-free soils with the FC-margin sites constituting a transitional environment (phase 2, model A). If the disturbance is continuous or multiple sequential disturbances occur over the lifespan of the FC, the FC microbial communities (margin and centre) would initially be determined by stochastic processes. Subsequently, a combination of niche-partitioning (margins constituting a buffered environment separating the well-defined vegetated and not impacted (control) environment and the plant-free FC centers) and stochastic processes (continuous disturbance) would be responsible for microbial community assemblies (phase 2, model B). Finally, as FCs die, i.e. disturbance has ceased, a neutral assembly processes would occur, leading to less variable edaphic microbial communities in the newly vegetation-covered soils (phase 3, models A and B). As the structures of the FC-center communities studied presented higher intra-variability than those of the margins and the more homogenous control soils, either the niche-partitioning is still occurring (model A) or FC-centers constitute ever-changing environments (model B).

## Conclusion/Perspectives

In ecology, patterns of diversity have been shown to be influenced by four major mechanisms/processes (selection, drift, speciation and dispersal) and by various species interactions (predation, competition and mutualism) [43]. Altogether, our results suggest that FC niche-specific surface edaphic microbial communities are ‘selected’. Such selection could originate from (i) habitat/niche-filtration (e.g., rhizospheric communities vs open soil communities; [39]) or (ii) be related to their adaptation to (an) environmental disturbance(s) [41], such as the presence of an active microbial phyto-pathogen [33], a continuous hydrocarbon gas emission [14], [20] or vegetation spatial self-organization [15], [16], [42].

We are yet unable to conclude whether bacteria and/or fungi are actually responsible for the appearance and/or maintenance of Fairy Circles in the Namib Desert. We suggest that more extensive metacommunity/biogeography studies on groups of FCs at different developmental stages [3], [44], [45] using meta’omic’ approaches [46], [47] could assist in characterizing the functional basis of FC ecosystems and in potentially identifying the agent(s) responsible for their formation. Such studies should be extended to deep FC soil horizons as it has been shown that (i) sub-surface FC soils are more anoxic than the surrounding vegetation-covered soils [14], (ii) that the abundance of culturable anaerobic bacteria is higher in FC soils [48] and (iii) that sub-surface FC soils accumulate water [11]. Moreover, to fully address the origin and functioning of Namib FC ecosystems, a comparative chemical (soil and gas) and (micro)biological analysis of ‘rocky’ and ‘sandy’ FC formations is recommended.

## Supporting Information

Figure S1 **Principal component analysis (PCA) plot of the normalized environmental variables measured.** Numbers refer to the respective Fairy Circles.
(TIF)

Table S1 **Results of two-way crossed ANOSIM tests based on Euclidean distance matrices from normalized soil chemistry measurements**. R: ANOSIM statistic; p: probability level. *: Significantly different (p <0.05).
(DOCX)
