# Supplementary figures and images for: Niche-Partitioning of Edaphic Microbial Communities in the Namib Desert Gravel Plain Fairy Circles
Source: PLoS One. 2014 Oct 3;9(10):e109539. doi: 10.1371/journal.pone.0109539 (PMC4184855; doi:10.1371/journal.pone.0109539)

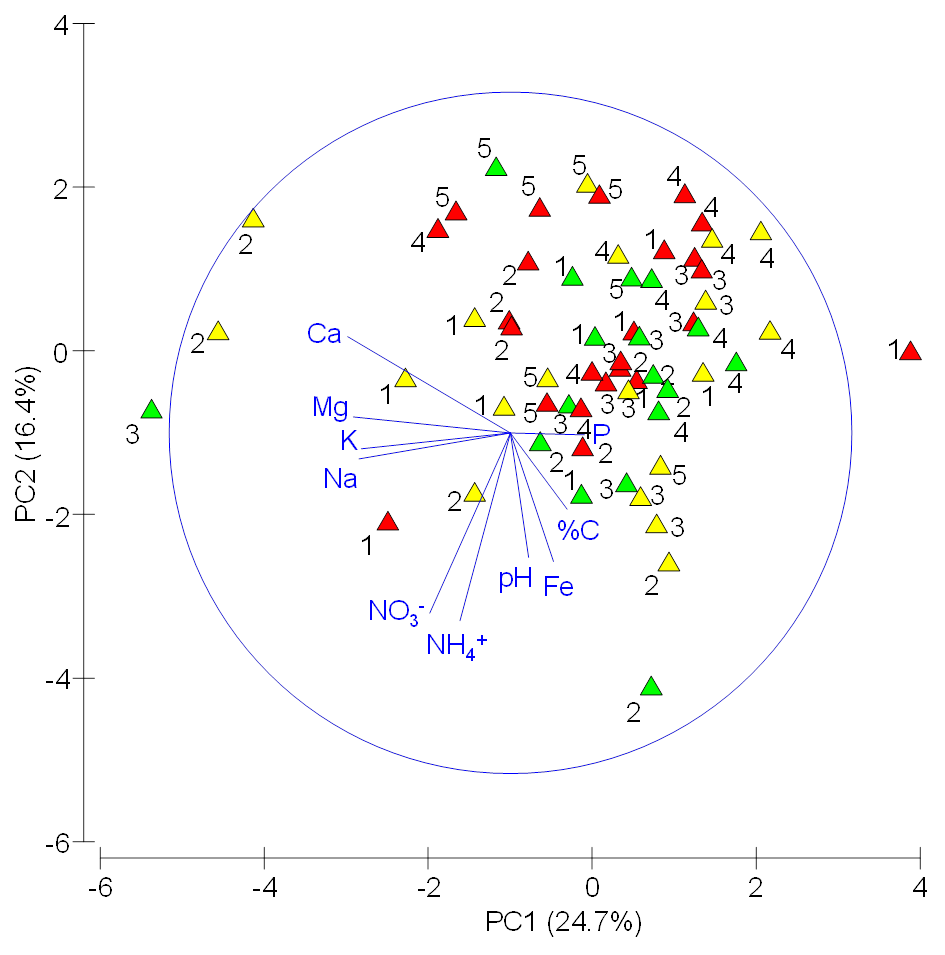

Supplement: Figure S1 — Principal component analysis (PCA) plot of the normalized environmental variables measured. Numbers refer to the respective Fairy Circles. (TIF) [file pone.0109539.s001.tif]
