# Supplementary material for: Niche-Partitioning of Edaphic Microbial Communities in the Namib Desert Gravel Plain Fairy Circles
Source: PLoS One. 2014 Oct 3;9(10):e109539. doi: 10.1371/journal.pone.0109539 (PMC4184855; doi:10.1371/journal.pone.0109539)
Supplement: Table S1 — Results of two-way crossed ANOSIM tests based on Euclidean distance matrices from normalized soil chemistry measurements. R: ANOSIM statistic; p: probability level. *: Significantly different (p <0.05). (DOCX) [file pone.0109539.s002.docx]

**Supplementary Table S1. Results of two-way crossed ANOSIM tests based on Euclidean distance matrices from normalized soil chemistry measurements**. R: ANOSIM statistic; p: probability level. *: Significantly different (p < 0.05).

|  | Differences among Fairy Circle Zones | |
| --- | --- | --- |
|  | R | p |
| Global Test | 0.144 | 0.003 * |
| Control vs Margin | 0.158 | 0.039 * |
| Control vs Centre | 0.149 | 0.029 * |
| Margin vs Centre | 0.153 | 0.021 * |
|  | Differences among Fairy Circles | |
|  | R | p |
| Global Test | 0.221 | 0.001 * |
| FC 1 vs FC 2 | 0.247 | 0.003 * |
| FC 1 vs FC 3 | 0.232 | 0.003 * |
| FC 1 vs FC 4 | 0.381 | 0.001 * |
| FC 1 vs FC 5 | 0.282 | 0.009 * |
| FC 2 vs FC 3 | 0.283 | 0.004 * |
| FC 2 vs FC 4 | 0.312 | 0.004 * |
| FC 2 vs FC 5 | 0.154 | 0.116 |
| FC 3 vs FC 4 | 0.243 | 0.005 * |
| FC 3 vs FC 5 | 0.172 | 0.062 |
| FC 4 vs FC 5 | 0.13 | 0.133 |
